# Procedures for Wavelength Calibration and Spectral Response Correction of CCD Array Spectrometers

**DOI:** 10.6028/jres.114.015

**Published:** 2009-08-01

**Authors:** A. K. Gaigalas, Lili Wang, Hua-Jun He, Paul DeRose

**Affiliations:** Biochemical Science Division National Institute of Standards and Technology, Gaithersburg, MD 20899

**Keywords:** CCD array spectrometer, fluorescence, spectral response correction, splicing procedure

## Abstract

This work describes a procedure for acquiring a spectrum of an analyte over an extended range of wavelengths and validating the wavelength and intensity assignments. To acquire a spectrum over an extended range of wavelengths with a spectrometer with a charge coupled device (CCD) array detector, it is necessary to acquire many partial spectra, each at a different angular position of the grating, and splice the partial spectra into a single extended spectrum. The splicing procedure exposes instrument dependent artifacts. It is demonstrated that by taking a spectrum of a reference irradiance source and making spectral correction, the artifacts exposed by the splicing are removed from the analyte spectrum. This is because the irradiance reference spectrum contains the same artifacts as the analyte spectrum. The artifacts exposed by the splicing depend on the wavelength of the splice; therefore it is important to measure the irradiance reference spectrum for the same range of wavelengths used to measure the spectrum of the analyte solution. In other words, there is no general spectral correction factor which is applicable to spectra taken for different range of wavelengths. The wavelength calibration is also carried out by splicing many partial spectra from a source like a krypton lamp. However the wavelength assignments are not sensitive to the splicing procedure and the same wavelength calibration can be used for spectra acquired over different extended wavelength ranges. The wavelength calibration checks the validity of the setting of the grating angular position, and the assignment of wavelengths to individual pixels on the CCD array detector. The procedure is illustrated by measuring the spectrum of an orange glass and the spectrum of a suspension of microalgae.

## 1. Introduction

Spectrometers with CCD array detectors are finding increased application in many fluorescence based measurements. The large data acquisition rates that are possible with these spectrometers permit greater sensitivity and spectrum acquisition speed. The major limitation of the array based spectrometers is the relatively narrow spectral range (≈ 60 nm for a spectrometer with a focal length of 0.25 m and a grating with groove density of 1200 mm^−1^) that can be covered during one acquisition of a spectrum. In many cases, fluorescence measurements require a spectral range of several hundred nm. For example the study of fluorescence resonant energy transfer (FRET) requires the coverage of the emission spectra of two fluorophores, microspheres used in multicolor cytometer calibration have fluorescence spectra extending over several hundred nm, and the fluorescence emission of many photosynthetic organisms (e.g., microalgae) have very large Stokes shifts. Acquisition of a fluorescence spectrum with an array based spectrometer over an extended spectral range requires the accumulation of several partial spectra. The objective of the present work is to present a procedure for combining (splicing) the partial spectra into a single extended spectrum. It is shown that artifacts exposed by splicing can be eliminated by performing a spectral response correction. The nature of the artifacts has been discussed in Ref. [1].

## 2. Spectrometers

A spectrometer with a CCD array detector measures the photon spectrum over the range of wavelengths that are subtended by the length of the CCD chip. The wavelength range that is observed is determined by the grating orientation which sets the wavelength of light incident on the central pixel of the CCD array detector. For example, setting the grating orientation of a spectrometer to “630 nm” directs the photons of wavelength 630 nm to fall upon the central pixel of the CCD array detector (e.g., the 512th pixel for a CCD with 1024 pixels). The pixels on both sides of the central pixel respond to wavelengths in the range 600 nm to 660 nm (given a grating with a groove density of 1200 mm^−1^) To obtain another range of wavelengths, the grating has to be rotated and another spectrum collected. To obtain an extended spectrum from 400 nm to 700 nm, it is necessary to rotate the grating 5 to 6 times and collect that number of different partial spectra. In contrast, a scanning spectrometer takes data over any extended spectral range by rotating the grating incrementally and allowing photons with a specified wavelength to reach the exit slit and the photomultiplier tube (PMT) detector mounted behind the exit slit. In this case, the position of the grating is always the same at a particular wavelength.

In order to validate the spectrum measured with any spectrometer, it is necessary to perform a wavelength calibration, and correct for spectral response of the spectrometer detector. In the case of a CCD array spectrometer, the task of wavelength calibration over an extended wavelength range requires the collection of many partial spectra from a source such as a krypton lamp. The same is true for the collection of a reference relative irradiance spectrum which is used for estimating spectral response. In both cases, there is a requirement for splicing multiple partial spectra into the final extended spectrum. The same splicing method is used to obtain the spectrum that is used for wavelength calibration, the spectrum for the estimate of spectral response, and the analyte spectrum. Therefore the artifacts exposed by the splicing method have to be considered as part of the overall budget associated with the uncertainties of the measured spectrum using a CCD array spectrometer. Scanning spectrometers, which collect data at a sequence of wavelengths each of which is associated with a different grating orientation, have many of the same instrumental artifacts. However, in the case of scanning spectrometers the data presentation does not expose these artifacts in such a graphic manner.

In the following, we use a monochromator with a CCD array detector to measure the spectra of a Kr calibration source and a irradiance reference source over the range of wavelengths 400 nm to 800 nm. The resulting extended spectra are used to calibrate the spectrometer wavelength and obtain a correction for spectral response. The emission spectra of orange glass and microalgae suspension are measured with 405 nm excitation. The same splicing method is used to assemble the extended glass and microalgae spectra. We discuss the uncertainties, and compare the measured algae spectrum with the spectrum obtained using a scanning spectrometer.

### 2.1 The Operation of a Spectrometer With a CCD Array Detector

**Fig. 1:**
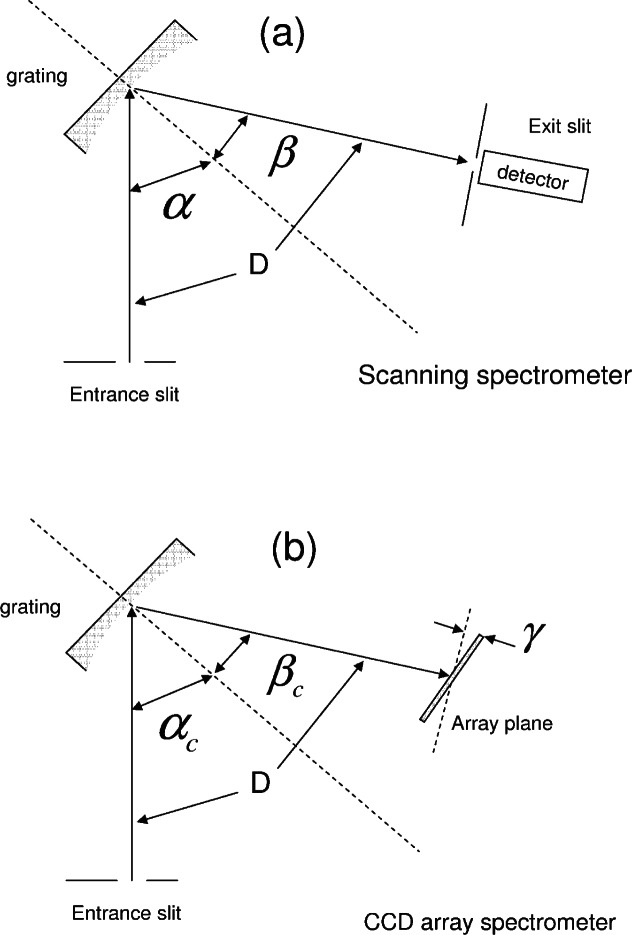
(a) The geometry of a scanning spectrometer. The angle denoted by D is fixed by the construction of the spectrometer. The angles denoted by *α* and *β* can be changed by rotating the grating (shaded shape) around the vertical (normal to the plane of the figure) line through the center of the grating. The values of *α* and *β* determine the wavelength of the light which reaches the exit slit of the spectrometer. (b) The geometry of the spectrometer with a CCD array detector. The angles *α_c_* and *β_c_* refer to the central pixel of the CCD array. The central pixel is similar to the exit slit in the scanning spectrometer. The other pixels in the CCD array detect light with wavelengths given by Eq. (2) in the text.

Figure 1a and Fig. 1b show the geometric relationship between the entrance slit, the grating, and the exit slit in a scanning spectrometer and a spectrometer with a CCD array detector respectively. The three angles in Fig 1a and Fig 1b and the properties of the grating determine the wavelength of the photons reaching the exit slit or the central pixel of the CCD array. The construction of the scanning instrument fixes the value of the angle D defined as the angle subtended by the entrance and exit slits at the center of the grating. Rays of light entering through the entrance slit make an angle of *α* with the normal to the grating surface (dashed line in Fig 1a) at the center of the grating. Similarly, light rays leaving through the exit slit make an angle of *β* with the normal. The grating is free to rotate around the axis perpendicular to the plane of Fig 1a and passing through the center of the grating. The three angles shown in Fig 1a are related by the equation *D* = *β* − *α*. (Note the sign convention for angles: counterclockwise rotation from the grating normal is positive and clockwise rotation is negative). The fundamental grating equation gives the relationship between the wavelength of light diffracted in the first order and the angles *α* and *β*

$$\lambda =\frac{\left(\sin(\alpha )+\sin(\beta )\right)}{n}\cdot 10^{6}$$ (1)

where *n* is the groove density. In the scanning instrument, the wavelengths of the light reaching the exit slit are selected by rotating the grating to appropriate values of the angles *α* and *β*. An angular encoder keeps track of the angular position of the grating and permits an accurate prediction of the expected wavelength exiting the instrument. The wavelength calibration of the instrument is performed by scanning through the grating angles and measuring a spectrum with known wavelengths. A comparison of the measured values of the wavelengths with the known values constitutes a wavelength calibration of the spectrometer. A similar procedure is carried out with a source of known spectral irradiance to determine the instrument spectral response.

Figure 1b shows the geometric arrangement in a spectrometer with a CCD array detector. Note that the exit slit is replaced by the central pixel of the CCD array. The angles *α_c_* and *β_c_* are associated with rays that hit the central pixel of the array and the wavelength of these rays is given by Eq. (1) as in the case of the scanning instrument. Rays that hit other pixels of the array are associated with other wavelengths. The relationship between the pixel number *i* on the CCD chip and the wavelength, *λ_i_*, is given by Eq. (2)[2]

$$\lambda_{i}=\frac{\left(\sin(\alpha_{c})+\sin(\beta_{\lambda_{i}})\right)}{n}\cdot 10^{6}$$ (2)

where *n* is the groove density of the grating (grooves per mm), 10^6^ is a conversion factor to give wavelength in units of nm, and the other factors in Eq. (2) are defined in Eq. (3) below using the notation given in Ref. [2].

$$\begin{array}{c} sum=2\arcsin(10^{-6}\cdot n\cdot \lambda_{c}\frac{1}{2\cos(D/2)})\\ \beta_{c}=\frac{D+sum}{2}\\ \alpha_{c}=\beta_{c}-D\\ HB_{c}=F\sin(\gamma )\\ L_{h}=F\cos(\gamma )\\ HB_{\lambda i}=HB_{c}+P_{w}(P_{i}-P_{c})\\ \beta_{\lambda i}=\beta_{c}+\gamma -\arctan(\frac{HB_{\lambda i}}{L_{h}}). \end{array}$$ (3)

Where *γ*, shown in Fig. 1b, is the inclination angle of the array plane relative to the plane which is normal to the line connecting the center of the grating and the central pixel of the array. *F* is the monochromator focal length in mm, *λ_c_* is the wavelength in nm of the light hitting the central pixel, *P_i_* is the pixel number, *P_c_* is the number of the central pixel, and *P_w_* is the pitch of the pixels in mm. For the system used in these measurements (see below), the parameter values as provided by the manufacturer are shown in Eq. (4).

$$\begin{array}{l} D=16.76{}^{\circ}\\ \gamma =2.4{}^{\circ}\\ F=270\;mm\\ P_{c}=512\\ P_{w}=26.5\times 10^{-3}\;mm\\ P_{i}=1\;\text{to}\;1014. \end{array}$$ (4)

A measurement of a spectrum is carried out by exposing the CCD array for a specified time and “reading” the contents of the individual pixels in the array. In order to assign a wavelength it is necessary to set a central wavelength as in the scanning instrument (see Eq. (1)) and to calculate the wavelength associated with each pixel of the array (see Eq. (2)). Since the CCD array has a finite dimension, the range of the measured wavelengths for a fixed central wavelength is limited. For the instrument used in these measurements, the wavelength range extended approximately 30 nm on either side of the central wavelength. Therefore to measure a spectrum over a larger range of wavelengths it was necessary to measure multiple partial spectra each associated with a different central wavelength. The multiple partial spectra have to be spliced into a single extended spectrum. The splicing algorithm becomes part of the measuring procedure. This work suggests that the wavelength calibration, the spectral correction, and the acquisition of the analyte spectrum should be performed using the same splicing procedure (central wavelengths of the partial spectra are the same in all cases).

## 3. Measurements With a Spectrometer With a CCD Array Detector

**Fig. 2:**
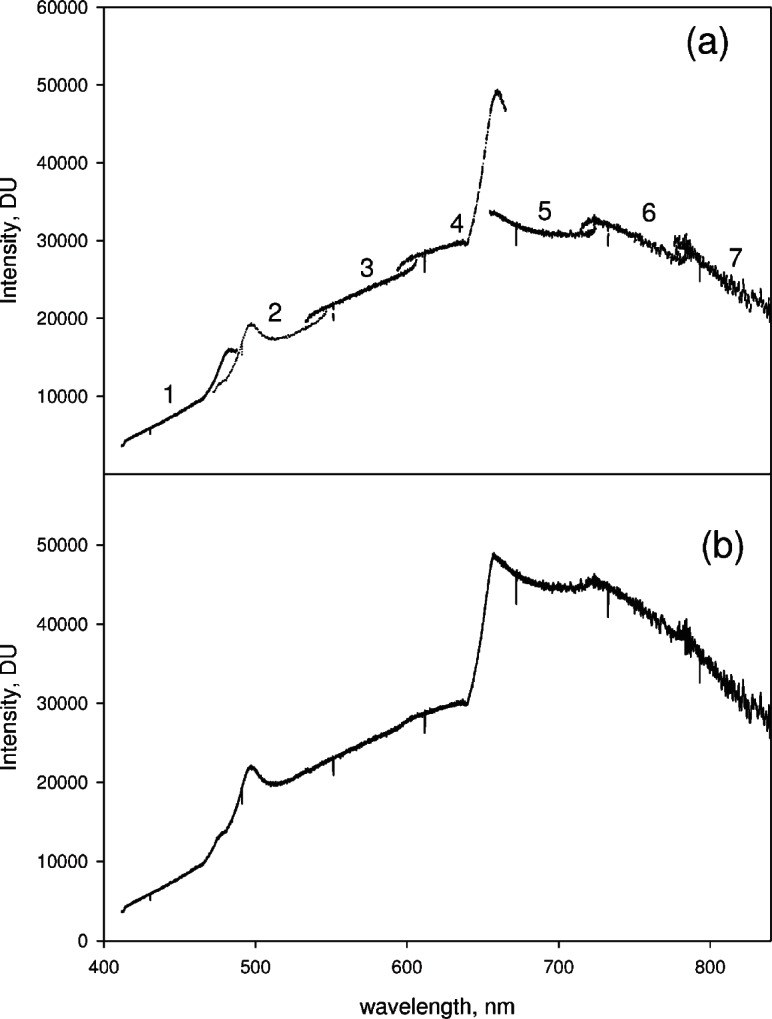
(a) The solid traces show seven partial spectra from a reference irradiance lamp taken with a CCD array spectrometer set at seven different central wavelengths. The regions near 490 nm and 660 nm exhibit different response for each of the overlapping partial spectra. The differences can be attributed to dependence of the grating efficiency on grating angle at the blaze angle (515 nm) and at the Wood’s anomaly (660 nm). (b) The solid trace shows the extended spectrum which was obtained by splicing the seven partial spectra shown in (a). The splicing was performed by requiring continuity at the midpoint of the overlap region between any two partial spectra. There are significant artifacts exposed by the splicing procedure. Figures 3 and 4 and the accompanying text discusses the splicing procedure and how to remove the artifacts exposed by splicing.

The spectrometer consisted of a 270M monochromator (HORIBA Jobin-Yvon, Inc., Edison, N,J)^1^ with a SpectraOne CCD detector (HORIBA Jobin-Yvon, Inc., Edison, N,J). Data was collected using LabView software modules supplied by HORIBA Jobin-Yvon. The partial spectra (PS) were collected with the slit width set to 0.2 mm, the integration time set to 100 ms, dark counts subtracted, and the central wavelength set to the appropriate value using a LabView module. The central wavelengths of the partial spectra were calculated to cover the specified wavelength range with an overlap between the partial spectra. The central wavelengths were set sequentially in ascending order, the partial spectra acquired, and the accumulated partial spectra were displayed for preliminary evaluation before transfer to a file. Figure 2a shows the 7 partial spectra from a calibrated reference lamp. The number above each partial spectrum gives its place in the sequence of accumulation. The lamp spectrum is shown because it gives a strong response over the entire wavelength range from 420 nm to 840 nm. The partial spectra were spliced and analyzed using Mathcad and SigmaPlot software applications. The splicing was accomplished by joining the partial spectra at a wavelength in the midpoint of the overlap region and adjusting the intensity of one of the partial spectra to match the intensity of the other. Splicing of some of the partial spectra (such as PS1 and PS2, and PS4 and PS5) exposed significant artifacts and distorted the extended spectrum as shown in Fig. 2b. The splicing procedure and the elimination of the exposed artifacts are discussed below.

### 3.1 Procedure for Splicing the Partial Spectra

**Fig. 3:**
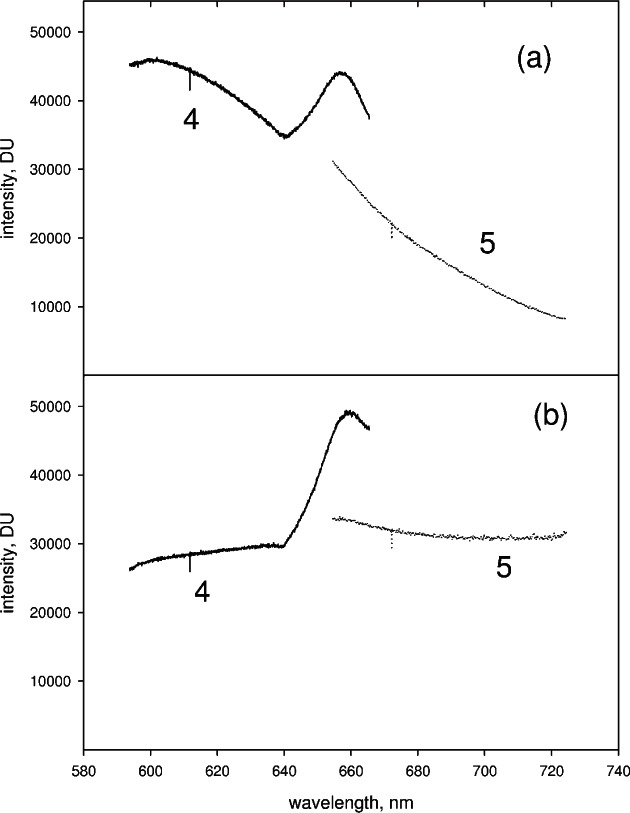
(a) Two partial spectra (PS4 solid trace and PS5 dotted trace) taken for a orange glass sample in the spectrometer (b) Two partial spectra (PS4 and PS5) taken for a reference lamp in the spectrometer. The artifacts introduced by the splicing procedure can be eliminated by correcting the orange glass spectrum shown in (a) for spectral response variation of the CCD detector obtained from the spectra of the reference lamp shown in (b). The results of the correction are shown in Fig. 4.

The splicing of the partial spectra was carried out pair wise starting with the partial spectra at lowest wavelengths. The same splicing procedure was carried out for all pairs of partial spectra. The splicing procedure will be illustrated using two specific partial spectra (PS4 and PS5) shown in Fig. 3a. The partial spectra in Fig. 3a were taken for an orange glass sample excited with 404 nm laser light. This case serves as an excellent example because the two partial spectra exhibit the largest intensity difference in the overlap region. Figure 3b shows two partial spectra (PS4 on the left and PS5 on the right) taken for the reference lamp. Both the glass spectra and the reference lamp spectra were collected using the same set of central wavelengths. In the following, we discuss the splicing procedure which is applied to the partial spectra taken for both the reference lamp and the orange glass sample. Finally it is shown how the reference spectrum is used to correct for spectral response and eliminate artifacts in the spectrum taken for the orange glass.

Equation 2 is used to assign wavelengths to each pixel in PS4 and PS5. The wavelength assignment is slightly different in the overlap region of the two partial spectra shown in Fig. 3a (and Fig. 3b). The wavelength assigned to the first pixel of PS5 is 654.574 nm, while the wavelength of the pixel in PS4 that best matches the initial wavelength in PS5 is 654.600 nm. The difference between the two “matching” wavelengths is 0.026 nm which is smaller than 0.070 nm, the interval between wavelengths assigned to adjacent pixels in either of the partial spectra. The match between wavelengths was obtained by simply taking the assigned wavelength of the first pixel in PS5 and comparing this wavelength to the assigned wavelengths of sequential pixels in PS4 starting at the last pixel. A match was obtained when the difference between the assigned wavelengths in the two partial spectra was less than 0.040 nm. The number of steps required to arrive at a wavelength match was divided by two and called “mid”. The value of “mid” was used to define the number of the pixels at which the two partial spectra were joined (spliced). The spliced spectrum consisted of PS4 from pixel 0 up to the pixel 1023-mid, and PS5 starting at pixel mid-1 and going to pixel 1023. A check was carried out to ensure that the wavelengths of the adjoining pixels in the two partial spectra were indeed adjacent (difference of wavelength < 0.05 nm, which may be slightly smaller than the spacing of the wavelength between pixels in any given partial spectrum).

After properly joining the wavelengths of the two partial spectra PS4 and PS5, the intensities of the two partial spectra were matched. As seen in Fig. 3a, the measured signal in the overlap region is very different for the two partial spectra. There are two wavelength regions where there is a large variation in the overlap region between two partial spectra. The region at about 490 nm corresponds to the blaze angle, and the region at 630 nm corresponds to the region of Wood’s anomaly [3].

**Fig. 4:**
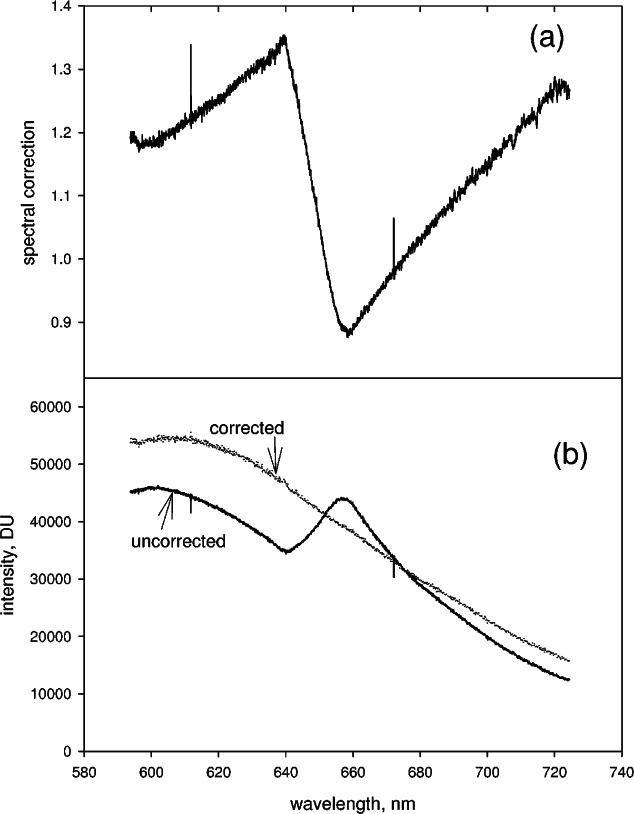
(a) The solid trace shows the spectral response correction factor. To obtain this trace, first, the partial spectra shown in Fig. 3b were spliced and normalized by dividing all of the values in the spliced spectrum by the value a 670 nm. Second, the calibrated irradiance values of the reference lamp were normalized by the value at 670 nm. The solid trace shows the normalized irradiance values divided by the values of the normalized spliced spectrum. This ratio constitutes the spectral correction factor. (b) The solid trace in Fig. 4b shows the spliced spectrum resulting from joining PS4 and PS5 in Fig. 3a. Multiplying the uncorrected spliced spectrum by the spectral correction factor, shown in (a), results in the corrected spectrum shown by the dotted line in Fig. 4b. The correction for spectral response eliminates artifacts exposed by the splicing procedure.

Figure 3b shows PS4 and PS5 acquired for the reference lamp. Clearly the splicing artifacts are also present in the reference spectrum. The spectral response correction is found by taking the ratio of the normalized calibrated radiance to the normalized measured reference spectrum. The normalization is relative to a value at some chosen wavelength in the entire spliced spectrum. Figure 4a shows the resulting spectral correction obtained after splicing the partial spectra shown in Fig. 3b. The spectral response correction contains the artifacts exposed by the splicing in the denominator, and upon multiplication with the measured analyte spectrum, the same artifacts in the measured analyte spectrum are eliminated. The corrected spliced spectrum obtained from the partial spectra in Fig. 3a and the spectral correction in Fig. 4a is shown by the dotted line in Fig 4b. The solid trace in Fig. 4b shows the uncorrected spectrum with the artifact exposed by the splicing at approximately 650 nm.

Special care has to taken when splicing two partial spectra with an overlap region having a small intensity. The noise in the signal contained in any one pixel is significant and normalizing the intensity of any two pixels may result in noise related artifacts. It is necessary to smooth the data near the splice and then match the two adjacent pixels using the smoothed data. The splicing problem associated with small intensities is encountered when splicing a Kr lamp spectrum. The wavelength regions between the spectral lines are background and have weak intensity signals. Since the background is flat, effects of noise can be reduced by simply averaging the signals of several pixels adjacent to the pixels used in the splicing.

### 3.2 Wavelength Calibration of a Spectrometer With a CCD Array

**Fig. 5:**
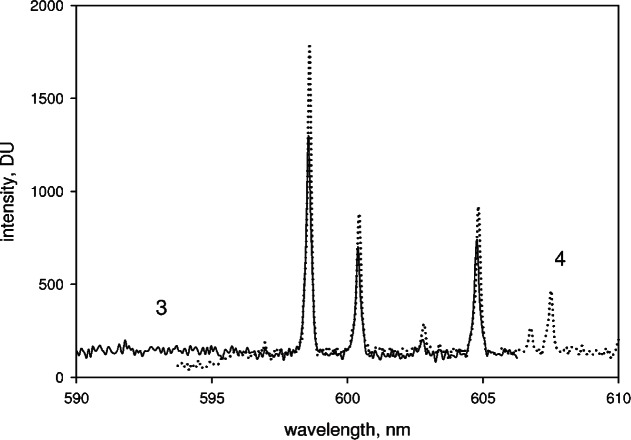
The overlap region of two partial spectra of Kr lamp taken by the CCD spectrometer. The solid trace shows the partial spectrum with the central wavelength set to 560 nm, and the dotted trace shows the partial spectrum taken with the central wavelength set to 630 nm. The values of the peak maxima in the overlap region differ by less than 0.1 nm. The spliced spectrum was obtained by joining the two spectra shown in Fig. 5 at the common wavelength of 600 nm. The exact procedure used in the splicing is described in the text.

The task is to combine the wavelength and intensity data in the overlap regions between partial spectra acquired for a Kr lamp in order to obtain a continuous spectrum over the extended range of wavelengths. Figure 5 shows the overlap region of measured partial spectra from a Kr lamp source for the central wavelength settings of 560 nm (solid line on the left) and 630 nm (dotted line on the right). The wavelength assignments for each partial spectrum were performed using Eq. (2). the values of the central wavelengths were taken from the algorithm based on Eq. (1) and provided by the manufacturer. The overlapping region of the two partial spectra in Fig. 5 shows that there are small wavelength differences and large intensity differences. In addition, the relative intensity changes of the background and peak values are very different. Furthermore, there is also a systematic decrease in intensity at the pixels near the end of all observed partial spectra. The data in Fig. 5 suggests that if the two responses are combined linearly, the resulting peaks in the overlap region would be slightly broadened with wavelength values corresponding to averages of the values in the two spectra. We chose to splice the spectra at a specified wavelength and require that the intensity is continuous (duscussed previously). The splice wavelength usually does not fall on a spectral peak. The peaks of the extended spectrum were defined as groups of pixels with signals that exceed 500 DU (digital units). The background was of the order of 170 DU. The peak wavelength was found by a weighted average of the wavelength assigned to the pixels in a peak

$$\lambda_{p}=\frac{\sum_{i-1}^{n}\lambda_{i}I_{i}}{\sum_{i-1}^{n}I_{i}}.$$ (5)

The symbol *n* stands for the number of pixels in a peak. The symbols *λ_i_* and *I_i_* are the wavelength and the intensity associated with the *i*th pixel in the peak. The first column of Table 1 gives the wavelengths determined using Eq. (5) of a Kr spectral lamp. The second column in Table 1 gives the width of the peak that was calculated using Eq.

$$\sigma_{p}={\left(\frac{\sum_{i-1}^{n}{\left(\lambda_{i}-\lambda_{P}\right)}^{2}I_{i}}{\sum_{i-1}^{n}I_{i}}\right)}^{1/2}={\left(\frac{\sum_{i-1}^{n}\lambda_{i}{}^{2}I_{i}}{\sum_{i-1}^{n}I_{i}}-\lambda_{p}^{2}\right)}^{1/2}.$$ (6)

**Fig. 6:**
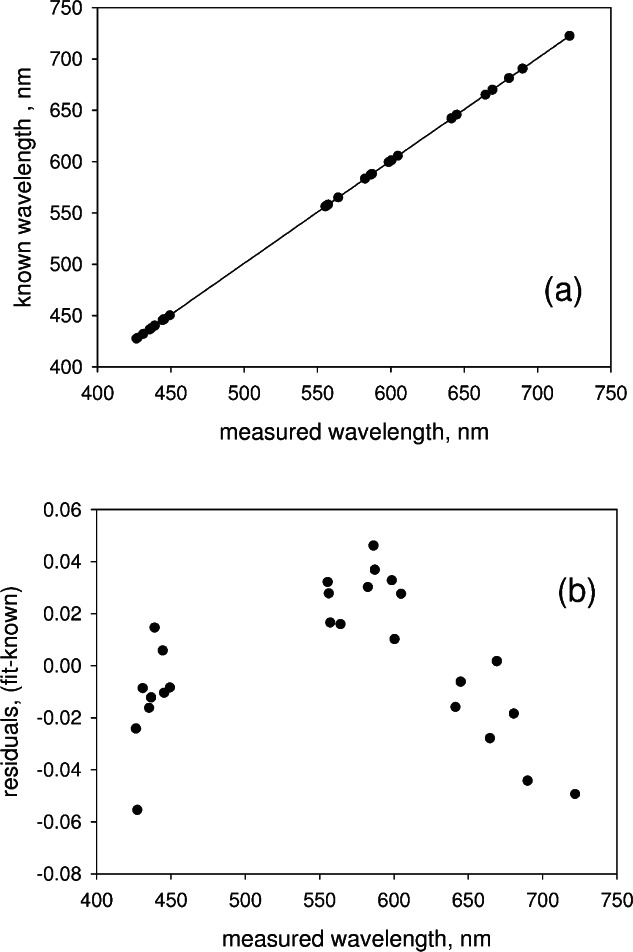
(a) The solid circles show the known wavelengths from an Kr lamp plotted as a function of the corresponding measured values (see Table 1). The solid line shows the fit to a linear function which constitutes a wavelength calibration of the spectrometer. (b) The solid circles show the difference between the known wavelengths and the best fit values as a function of the measured wavelengths. There is a quadratic trend in the deviations, however there is no obvious signature associated with the splicing regions.

Some of the weaker peaks gave a small width because only a few pixels had signals exceeding the threshold. Some of the stronger single peaks had a width of about 0.13 nm, a number which is indicative of the uncertainty in wavelength determination. Column 3 in Table 1 gives the known values of the wavelength [4,5]. The solid circles in Fig. 6a show a plot of the data in Table 1, and the solid line in Fig. 6a is a linear fit to the data. The linear fit constitutes a wavelength calibration of the CCD based spectrometer. The solid circles in Fig. 6b show the deviations between the values of the linear fit and the known wavelengths. It can be concluded from Fig. 6b that the wavelength calibration gives wavelengths accurate to ± 0.1 nm. In addition, Fig. 6b shows that while the residuals have a quadratic trend, there appears to be no systematic shifts associated with the splicing regions.

### 3.3 Spectral Response Correction

The spectral response correction, discussed in detail in the previous section on the splicing procedure, is needed to remove splicing artifacts. It is worthwhile to emphasize that the measurement of extended spectra, which requires the splicing of many partial spectra, needs spectral response correction to eliminate artifacts exposed by the splicing procedure. Thus spectral response correction is an integral part of the measurement.

### 3.4 Orange Glass Spectrum

**Fig. 7:**
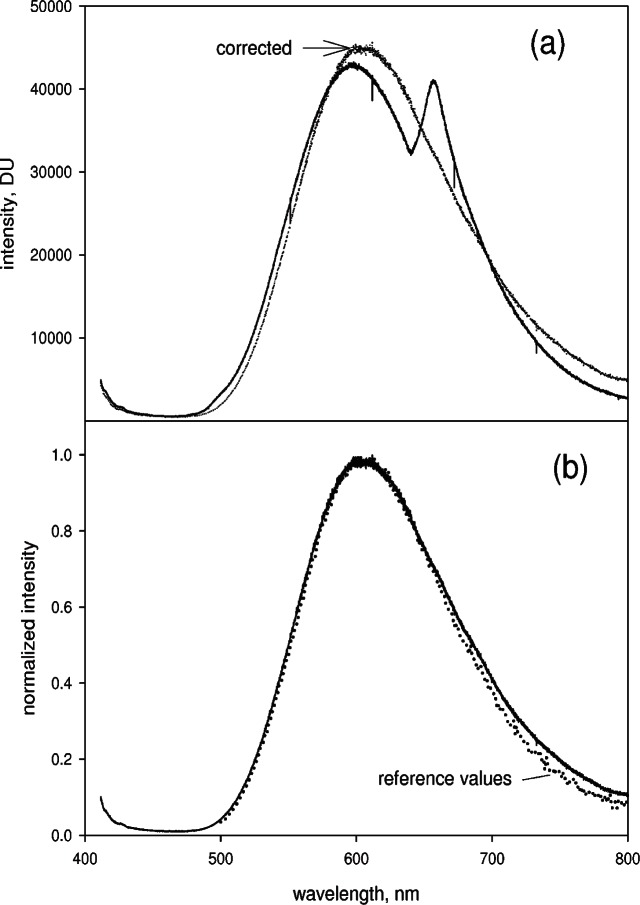
(a) The solid trace shows the measured extended spectrum from a SRM 2490 which is a piece of orange glass shaped in the form of a cuvette for easy placement in the spectrometer sample holder. The excitation was a 405 nm laser beam. The extended spectrum was obtained by splicing 7 partial spectra taken under the same conditions as the partial spectra shown in Fig. 2. The “bump” near 670 nm is an artifact exposed by the splicing procedure. The dotted trace shows the orange glass spectrum after subtraction of the background and correction for relative spectral response using the reference lamp as the irradiance standard. (b) The solid trace reproduces the corrected spectrum from part (a) and the dotted trace shows the normalized certified values of the relative emission spectrum from SRM 2490. The two normalized spectra diverge slightly beyond 640 nm. The discrepancy could be due to the different geometry of the two reference sources.

The solid trace in Fig. 7a shows the uncorrected spliced extended spectrum taken for a orange glass sample in the cuvette holder. The splicing was per formed as described above. The dotted trace in Fig. 7a shows the resulting corrected spectrum which accounts for the variation of the spectral efficiency of the CCD detector as well as removes artifacts exposed by splicing of the partial spectra. The reference source was the calibrated irradiance lamp. The resulting corrected spectrum is free from artifacts exposed by the splicing method. The orange glass sample is a NIST standard reference material 2490, SRM 2490, which has a certified relative emission spectrum for excitation at 412 nm. Since the standard was excited at Ex = 404 nm in this work, a new set of reference values for this excitation wavelength had to be calculated by comparing its corrected spectrum for Ex = 404 nm to its certified spectrum at Ex = 412 nm. The dotted trace in Fig. 7b shows the reference emission spectrum of SRM 2490 normalized to the maximum value. The solid trace in Fig. 7b is the normalized corrected spectrum from Fig. 7a. The correspondence between the two normalized spectra is excellent for wavelengths less then 650 nm. The small divergence at larger wavelengths is most likely due to the difference in source geometry relative to the sample. The reference lamp, which was used to obtain the spectral response correction is an extended source, while the orange glass spectrum was taken for a line source (the laser beam is focused inside the cuvette). In making spectral corrections it is best that the reference source and the analyte source have the same geometry. Therefore, SRM 2490 is an excellent reference source for making spectral correction to spectra obtained with a focused, continuous laser illumination.

### 3.5 Microalgae Spectrum

**Fig. 8:**
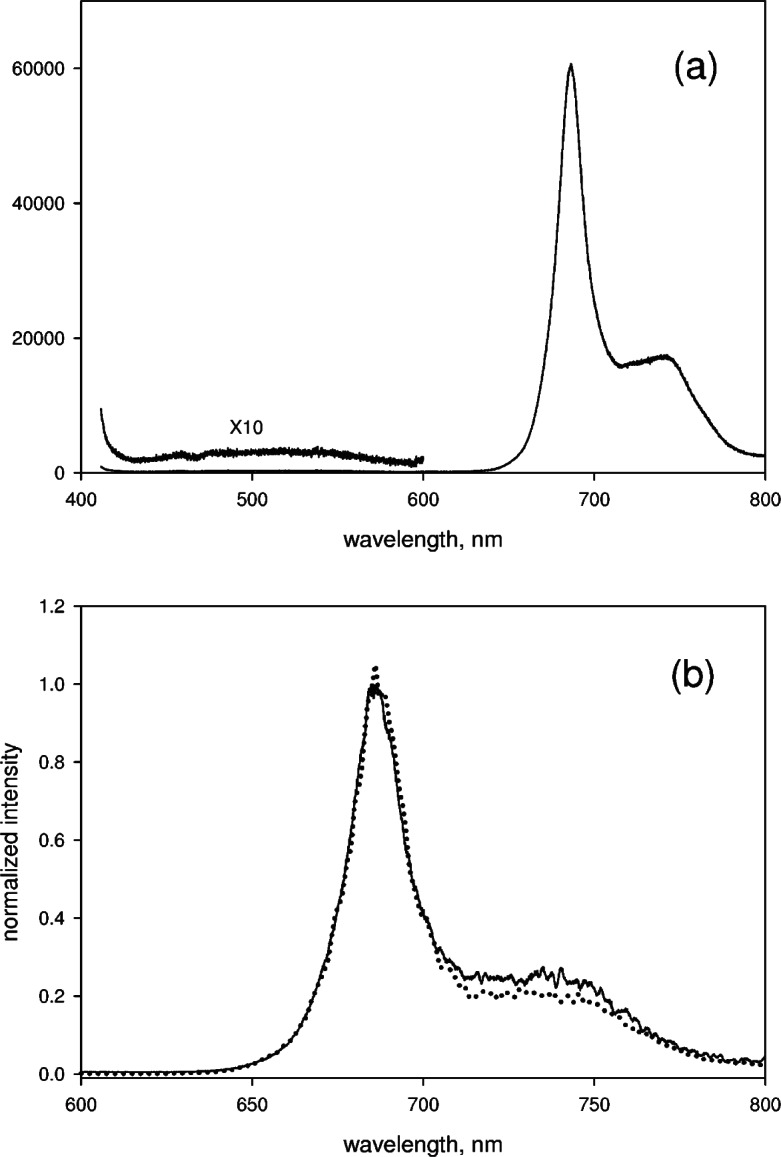
(a) The solid trace shows the measured signal from a cuvette filled with a suspension of microalgae and irradiated with a 404 nm laser line. The spectrum was corrected for background and for relative spectral response. The background was measured separately using a cuvette filled with the growth medium and the spectral response correction was determined using a reference lamp. The inset shows a portion of the spectrum magnified 10 times. The increase in the intensity at 410 nm is due to scattering from the microalgae. (b) The solid trace shows the emission spectrum from the algal suspension with background correction and spectra response correction using the SRM 2490 orange glass. The dotted lines show the emission spectrum from the algal suspension measured on a scanning spectrometer. The slight difference at 740 nm could be due to different illumination in the two instruments (discussed in text). The uncertainty in the measured normalized intensity was about 0.02 (a value obtained from the fluctuations of the normalized intensity).

The solid trace in Fig. 8 shows the final corrected fluorescence spectrum from microalgae. The spectrum in Fig. 8 was obtained by first subtracting the spectrum obtained for the growth medium without microalgae, and then correcting the subtracted spectrum for the relative spectral response using the reference lamp as the source. The inset in Fig. 8 shows the spectrum at lower wavelengths magnified 10 times. The corrected spectrum in Fig. 8 allows the calculation of a relative quantum yield of photons emitted between 410 nm and 630 nm (background) and the photons emitted between 631 nm and 804 nm (chlorophyll peak). The relative quantum yield, determined by summing the intensity over the specified wavelength ranges, is less than 0.015. It is remarkable that the collection of chlorophyll molecules in microalgae can equilibrate over such a large energy interval in the excited state with such negligible radiative decay. The solid trace in Fig. 8b shows the spectrum from microalgae with the spectral correction performed with SRM 2490 line source. The two microalgae spectra in Fig. 8a and Fig. 8b (normalized to the maximum value) show small discrepancies (not shown in the figure) at larger wavelengths. These discrepancies most likely originate from the differences in the reference source geometry. The dotted trace in Fig. 8b shows the normalized emission spectrum from the same micro-algae suspension acquired on a scanning spectrometer (Horiba Jobin-Yvon Fluorolog). There is a small discrepancy around 740 nm. Since both spectra were corrected for spectral response using SRM 2490, the discrepancy is most likely not instrumental. A likely source of the discrepancy is a dependence of the emission spectrum on the level of illumination [6]. The fluorescence emission originates from two photosynthetic systems I and II with the emission of photosynthetic system I occurring at larger wavelengths. Illumination at 404 nm can affect the two photosynthetic systems in a different manner resulting in different relative emission. Careful correction of the measured spectrum for spectral response (and splicing) makes the spectral band shape a meaningful physical variable.

## 4. Conclusion

A spectrometer with a CCD array detector accumulates a spectrum over an extended wavelength range by accumulating many partial spectra. The partial spectra have to be spliced to obtain the extended spectrum. The splicing procedure exposes artifacts which can be removed by correcting the extended spectrum for the variation of the spectral response of the CCD detector. The spectrum correction is implemented by measuring the spectrum of a reference source with known relative spectral irradiance (spectral shape). The known normalized spectral shape is divided by the normalized measured reference spectrum to obtain the relative spectral response correction. Multiplying the measured analyte spectrum by the spectral response correction eliminates the artifacts exposed by splicing. Wavelength calibration requires the measurement and splicing of partial spectra from a krypton (or equivalent) lamp. Comparison of the measured wavelengths and the known wavelengths suggests that artifacts exposed by splicing do not play a critical role in the wavelength calibration. Spectral response correction is best performed with a reference sources that has the same geometry as the analyte source. SRM 2490 is an excellent reference source for spectra acquired with a 404 nm laser line.

## Figures and Tables

**Table 1:** Comparison of known wavelengths (Ref. [5]) and wavelengths determined with the spectrometer with the CCD array

Measured wavelength* nm	Peak width nm	Known wavelength Nm
426.5	0.12	427.39
427.4	0.10	428.29
431.1	0.13	431.95
435.4	0.11	436.26
436.7	0.13	437.61
439.1	0.06	440.00
444.5	0.12	445.39
445.5	0.13	446.36
449.4	0.13	450.23
555.4	0.12	556.22
556.2	0.12	557.02
557.2	0.04	558.03
564.1	0.04	564.95
582.5	0.04	583.28
586.3	0.13	587.09
587.2	0.10	587.98
598.6	0.07	599.38
600.4	0.04	601.20
604.8	0.05	605.61
641.4	0.05	642.10
644.9	0.09	645.62
664.5	0.04	665.22
669.2	0.03	669.92
680.6	0.03	681.31
689.8	0.08	690.46
721.8	0.08	722.41

* the uncertainty of the wavelength measurement is of the order of 0.13 nm. This value is the width of intense single peaks.

## Footnotes

1 Certain commercial equipment, instruments, and materials are identified in this paper to foster understanding. Such identification does not imply recommendation or endorsement by the National Institute of Standards and Technology.
